# Designing Trojan Detectors in Neural Networks Using Interactive Simulations

**DOI:** 10.3390/app11041865

**Published:** 2021

**Authors:** Peter Bajcsy, Nicholas J. Schaub, Michael Majurski

**Affiliations:** 1 Information Technology Laboratory, National Institute of Standards and Technology (NIST), 100 Bureau Drive. Gaithersburg, MD 20899;; 2 National Center for Advancing Translational Sciences (NCATS), National Institutes of Health (NIH), Axle Informatics, 6116 Executive Blvd Suite 400, Rockville, MD 20852;

**Keywords:** neural network models, trojan attacks, security

## Abstract

This paper addresses the problem of designing trojan detectors in neural networks (NNs) using interactive simulations. Trojans in NNs are defined as triggers in inputs that cause misclassification of such inputs into a class (or classes) unintended by the design of a NN-based model. The goal of our work is to understand encodings of a variety of trojan types in fully connected layers of neural networks. Our approach is (1) to simulate nine types of trojan embeddings into dot patterns, (2) to devise measurements of NN states, and (3) to design trojan detectors in NN-based classification models. The interactive simulations are built on top of TensorFlow Playground with in-memory storage of data and NN coefficients. The simulations provide analytical, visualization, and output operations performed on training datasets and NN architectures. The measurements of a NN include (a) model inefficiency using modified Kullback-Liebler (KL) divergence from uniformly distributed states and (b) model sensitivity to variables related to data and NNs. Using the KL divergence measurements at each NN layer and per each predicted class label, a trojan detector is devised to discriminate NN models with or without trojans. To document robustness of such a trojan detector with respect to NN architectures, dataset perturbations, and trojan types, several properties of the KL divergence measurement are presented. For the general use, the web-based simulations is deployed via GitHub pages at https://github.com/usnistgov/nn-calculator.

## 1. Introduction

The problem of detecting trojans in neural networks (NNs) models has been posed in the Trojan in Artificial Intelligence (TrojAI) challenge [1] by the Intelligence Advanced Research Projects Agency (IARPA). For Rounds 1–4 of the TrojAI challenge, trojans in NNs are defined as triggers (local polygons or global filters) in input traffic sign images that cause misclassification of the input traffic sign class into another traffic sign class (or classes). When the poisoned NN-based model with trojan is used for inferencing, a user will not know about the introduced misclassification by adversaries unless the input for inferencing is presented with the trojan. With the widespread use of neural networks in life-critical applications, such as self-driving cars, the design of trojan detectors in NNs is driven by commercial and government agencies due to security concerns.

**Figure 1.**
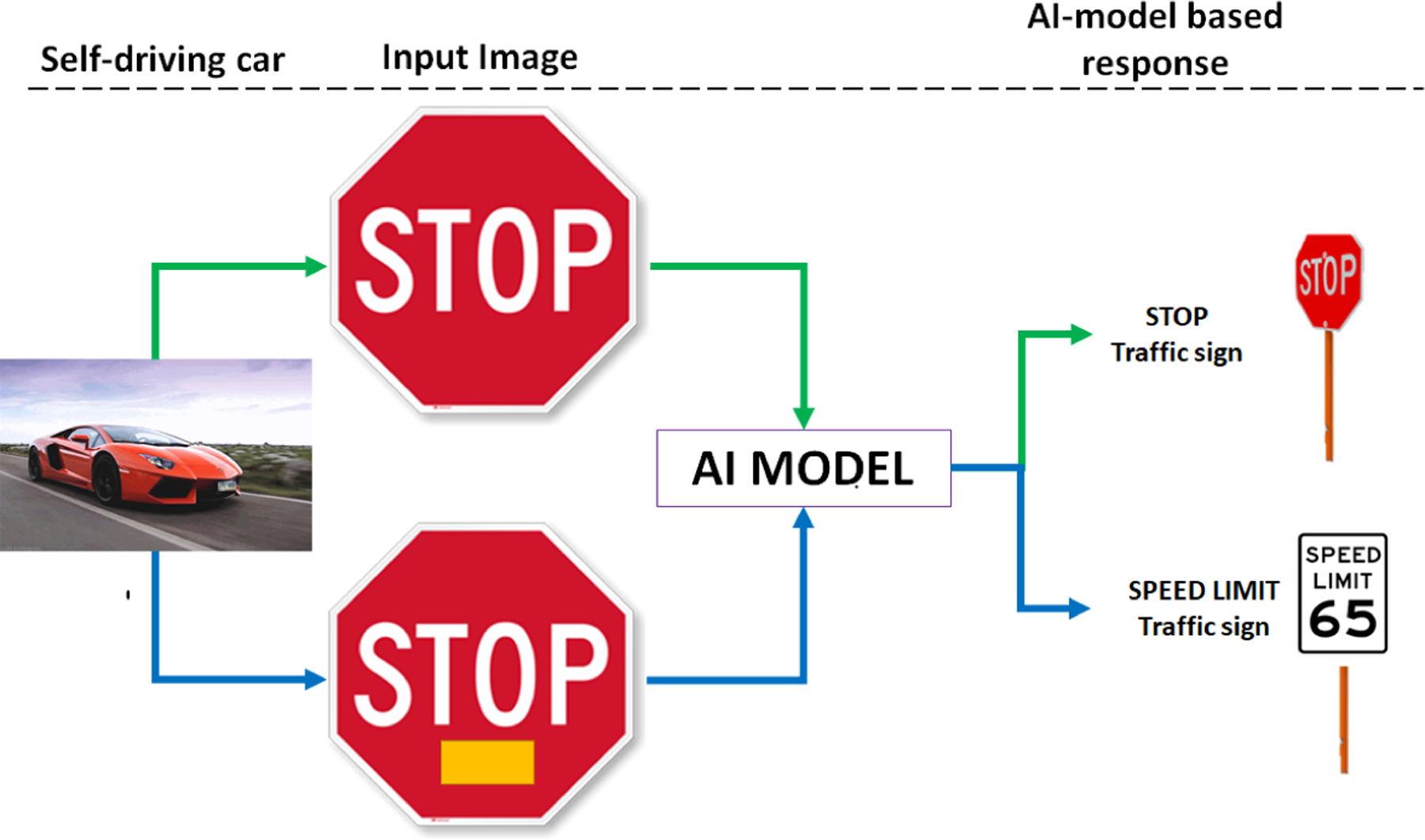
Trojan problem for traffic sign classification.

Figure 1 illustrates the problem of traffic sign classification with and without a trojan. An adversary with access to training data could embed some trojans into the training collection. For example, a yellow region added to the stop sign in Figure 1 will change the classification outcome of the stop sign into a speed limit sign. The yellow region is considered as a trojan (or trigger) embedded in a stop sign region which will re-assign the images with trojan from class A (stop sign) to class B (speed limit 65). Addiitonal information about simulating trojans and injecting trojans into images in TrojAI challenge datasets can be found in Appendix A.

The requirements on such detection solutions are multi-faceted since trojan detectors must achieve satisfactory performance for any NN-based task, any NN architecture, any type of trojan, any type of trojan detection input, and under limited computational time and constrained hardware specifications. Our work is motivated by the need to gain basic insights about trojans, their interactions with NNs, and NN measurements that can indicate the presence of trojans. This work aims at providing an interactive simulation environment for (a) gaining such insights and (b) assessing the difficulty of detecting several trojan types.

We address three specific problems in the aforementioned context. The first problem is in creating an interactive simulation environment for quick evaluations of (1) NN models with varying complexities and hyper-parameters, (2) datasets with varying manifold representation complexities and class balance ratios, and (3) measurements based on varying approaches and statistical analyses. The second problem lies in designing NN efficiency measurements with understood sensitivity to variations in NN architectures, NN initialization and training, as well as dataset regeneration. The third problem is in devising an approach to detecting trojans embedded in NN models.

The problems come with associated challenges. The first challenge lies in the interactivity requirement. As of today, DL NN architectures are very complex; from 60K parameters in LeNet [2], to common networks having millions and billions of parameters (160 billion reported in [3]). Modern networks require hours or days to train on advanced graphics processing unit (GPU) cards [4]. The challenge of the second problem lies in the lack of explainable artificial intelligence (AI) [5] and AI mathematical models [6], [7], and [8]. The last challenge lies in the large search space of possible trojans, training data, DL NN architectures, and NN training algorithms that must be understood (see Section 2 for additional references).

Our approach is (1) to simulate nine types of trojan embeddings into dot patterns, (2) to devise measurements of NN states, and (3) to design Trojan detectors in NN-based classification models. The interactive simulations are built on top of TensorFlow Playground[9] and enable users to embed trojans into dot patterns, and perform storage and algebraic operations on datasets and NNs. As one part of the simulations, histograms of NN activities at each node and over each NN layer are computed as data inputs pass through the network (e.g., nodes/neurons are firing or not). These histogram distributions of activities at nodes and layers are visualized during simulations and used for deriving NN efficiency metrics. Efficiency of a NN model is understood as the utilization of all states available at each node and in each layer. For designing a trojan detector, it is assumed that NNs trained with trojans (TwT) have a higher efficiency than NNs trained without trojans (TwoT) because encodings of trojans requires engaging additional states.

The novelties of the work lie in:

- extending TensorFlow Playground [9] into a trojan simulator for the AI community,
- designing a Kullback-Liebler (KL) divergence based measurement of NN inefficiency,
- devising an approach to detecting embedded trojans in AI models based on KL divergence.

First, the authors conceived the concept of interactive neural network calculator in which (a) operands are 2D data and neural networks, (b) memory operations follow the operations provided by standard calculators (MC, MR, M+, M−, MS, AVG), (c) NN and data operators are applicable functions to design, parametrize, train, infer, and analyze (inefficiency, sensitivity) NN-based models, and (d) display of NN, data, and results is delivered in scrollable views of web browsers. In comparison to previous work, this is an extension to the Tensorflow Playground visualization developed in [9] for fully connected layers at small scale with additional constructed features and all NN calculator functionalities. Second, the authors designed a modified KL divergence measurement of NN states based on the parallels with information theory and based on computational cost considerations. In comparison to previous work, the modified KL divergence measurement is an extension to the NN efficiency and expressiveness concepts in [10] and [11]. Finally, the authors devised a methodology for trojan detection by investigating the simulations of multiple types of embedded trojans. In comparison to previous work, the trojan detection approach is an extension of the observation in [12] about pruned NNs having a higher resilience against adding malicious triggers. Thus, two identical models, one with and one without embedded trojan, will have different inefficiency/utilization measured by the modified KL divergence.

The theoretical contribution is in having a well-defined measurement for assessing efficiency of NN models. The practical implications lie in the fact that the documented simulations in this paper and many other simulations can be used for educational and research purposes. Such simulations contribute to advancing explainable AI concepts by the AI community.

## 2. Related Work

The problem of trojan detection in NNs has many variations based on what information and computational resources are available for trojan detection (type of attack, type of model architecture, model coefficients, training data subsets, description of trojans, number of classes to be misclassified by embedding trojans, classes that are misclassified by trojans, models that have been trained with trojans, computational complexity limits imposed on the delivered solution, etc.). The Rounds 1–4 of IARPA TrojAI challenge [1] are characterized by an increasing number of variations while keeping the focus on traffic sign image classification task. Other challenges related to TrojAI have already been posed, for example, the Guaranteeing AI Robustness against Deception (GARD) challenge [13]. As of today, none of the challenges can be quantitatively described in terms of their difficulty level which motivates our work.

In the previous work, the problem of trojans in AI has been reported from the view point of detecting trojans [14] [15], constructing trojan attacks [16], defending against trojans [17], and bypassing trojan detectors [18]. The problem of trojan presence is often related to the efficiency (or utilization) of DL NNs as introduced in the early publications about optimal brain [19] and optimal brain surgeon [20]. A few decades later, the topics of pruning links and trimming neurons are being explored in [21], [22], and [23] to increase an efficiency of Deep Learning (DL) NNs and to decrease NN model storage and computational requirements of model training. Our work is motivated by the past concepts of NN efficiency. However, our goal is to explore the hypothesis that NN models trained with trojans will demonstrate higher efficiency/utilization of NN than NN models trained without trojan. This hypothesis can be explained by the observations that encoding n predicted classes plus trojan will likely require a model with higher modeling capacity than encoding n predicted classes. One can illustrate this observation on the last layer of fully connected layers. If the last layer consists of one node, then the node output can discriminate only two classes. In order to discriminate/predict more than two classes, one must increase the modeling capacity to more nodes per layer. In comparison to previous work, our model efficiency-based approach is focused on reliable measurements in the context of trojan detection and is investigating questions about where trojans are encoded. We assume that the models TwoT and TwT are neither under-fitted nor over-fitted [24].

The problem of gaining insights about DL NNs has been approached by (1) mathematical modeling [6] (network layers), [7] (activation functions), [8] (wavelets), (2) feature and network visualizations [25] (across layers), [26](higher layers), [27] (discriminative features),[9] (fully connected layers at small scale), and (3) limited numerical precision of modeling to achieve ‘interactive’ response [28](quantized NN for mobile devices), [29] (binary weights for ImageNet), [30] (tradeoffs), [31] (binary NNs). Many insights are pursued with respect to representation learning [32], expressiveness [33], [10], and sensitivity and generalization (under- and over-fitting NN models) [34], [35]. From all past work, we leveraged the mathematical framework in [6], visualization called Tensorflow Playground in [9], and efficiency and expressiveness concepts in [10] and [11].

## 3. Methods

### 3.1. Trojan Simulations

**Figure 2.**
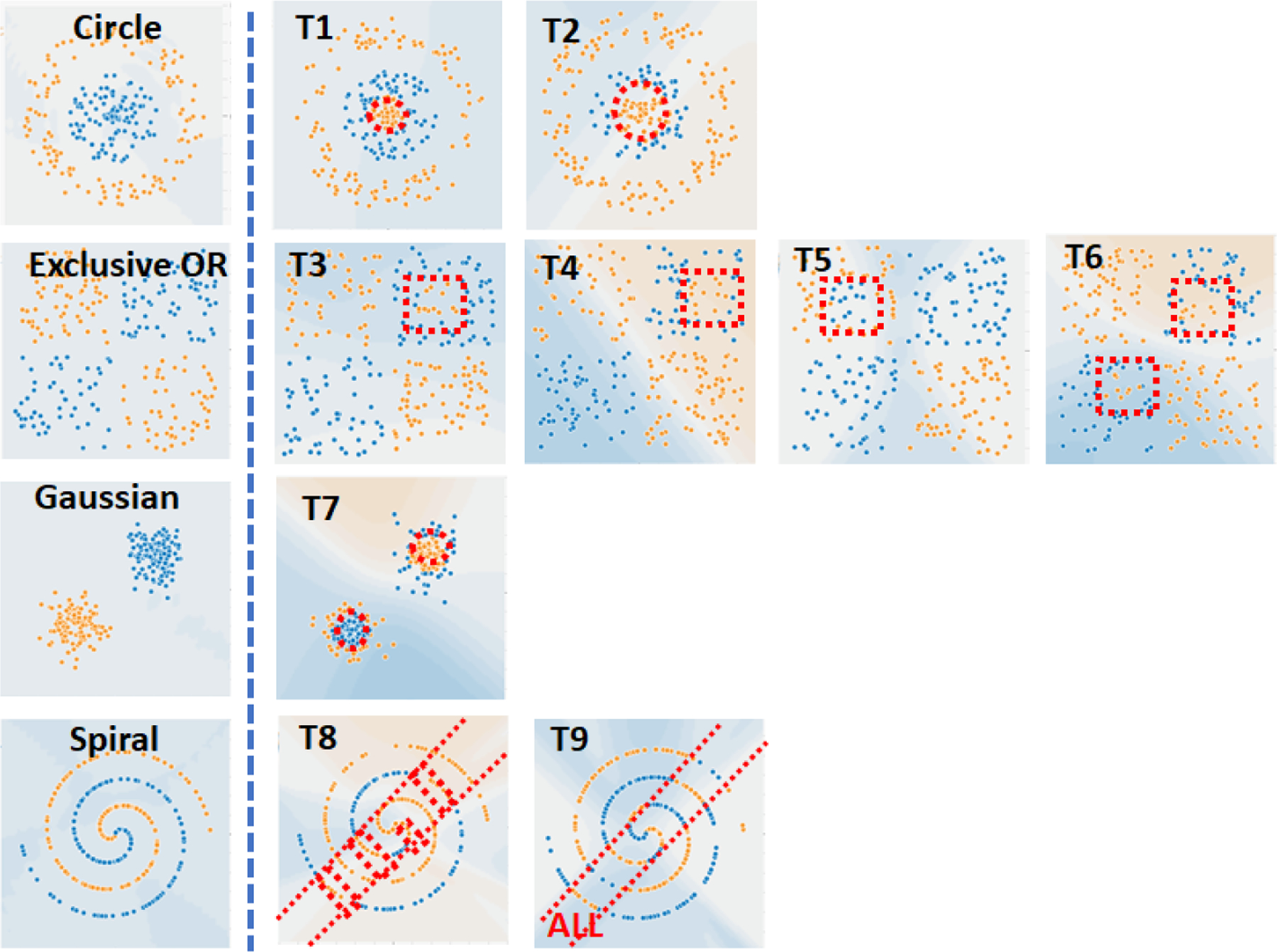
Illustration of nine trojan embeddings in four datasets. Orange dot - class 1, blue dot - class 2, red boundary encloses dots that represent a trojan embedding.

Our objective is to understand how the characteristics of trojans affect trojan detection, i.e. the discrimination of models trained without trojan (TwoT) and trained with trojan (TwT). In order to meet this objective, generators of nine types of trojans are created in the extension of TensorFlow Playground. Trojan embedding characteristics are generalized and described by (1) number of trojans per class, (2) number of trojans per contiguous region, (3) shape, (4) size, and (5) location of trojans inside of a class region. Figure 2 illustrate the nine trojan embeddings. Table 1 in Appendix B includes details about each trojan embedding.

**Figure 3.**
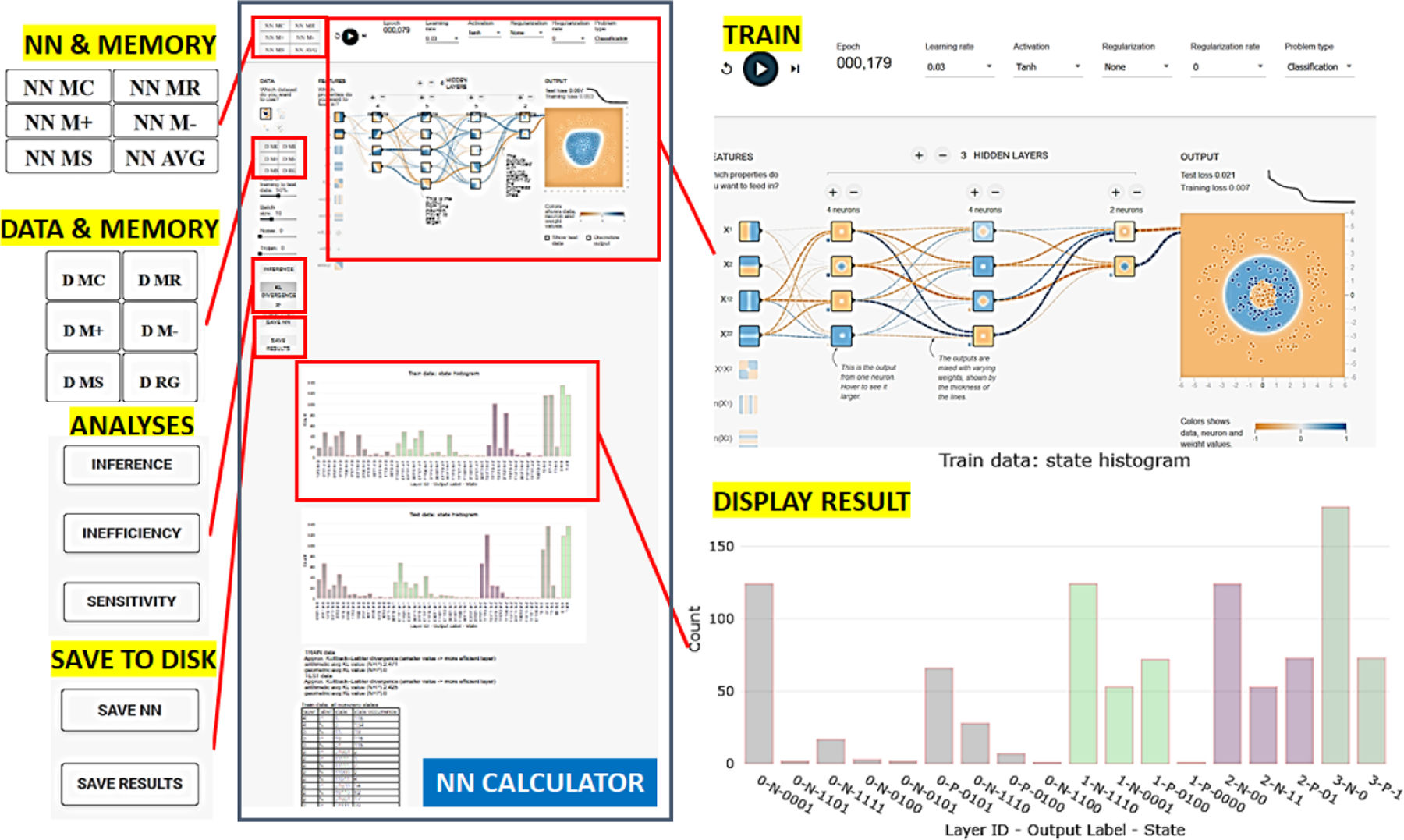
User interface for trojan simulator.

Once a trojan is embedded in a dot pattern, one needs to simulate training and inference using models TwoT and TwT. We extended TensorFlow Playground to enable operations on datasets and NN coefficients similar to the operations in a scientific calculator. We reused the symbols for MC, MR, M+, M−, and MS for clearing, retrieving, adding, subtracting, and setting memory with datasets (training and testing sets) and NN coefficients (biases and weights). The user interface is shown in Figure 3 (top left and middle left) where the standard five symbols are preceded with NN or D to indicate whether the operation is applied to NN or data. In addition, NN model averaging and dataset regeneration are included in order to study variability over multiple training sessions and random data perturbations. Evaluating combinations of datasets and NNs in real time enables one to explore full factorial experiments for provided factors.

### 3.2. Design of Neural Network Measurements

In this section, a NN inefficiency measurement is introduced from a histogram of NN states at each layer by using (1) KL divergence, (2) a reference state distribution, and (3) computational constraints.

#### States of Neural Network:

**Figure 4.**
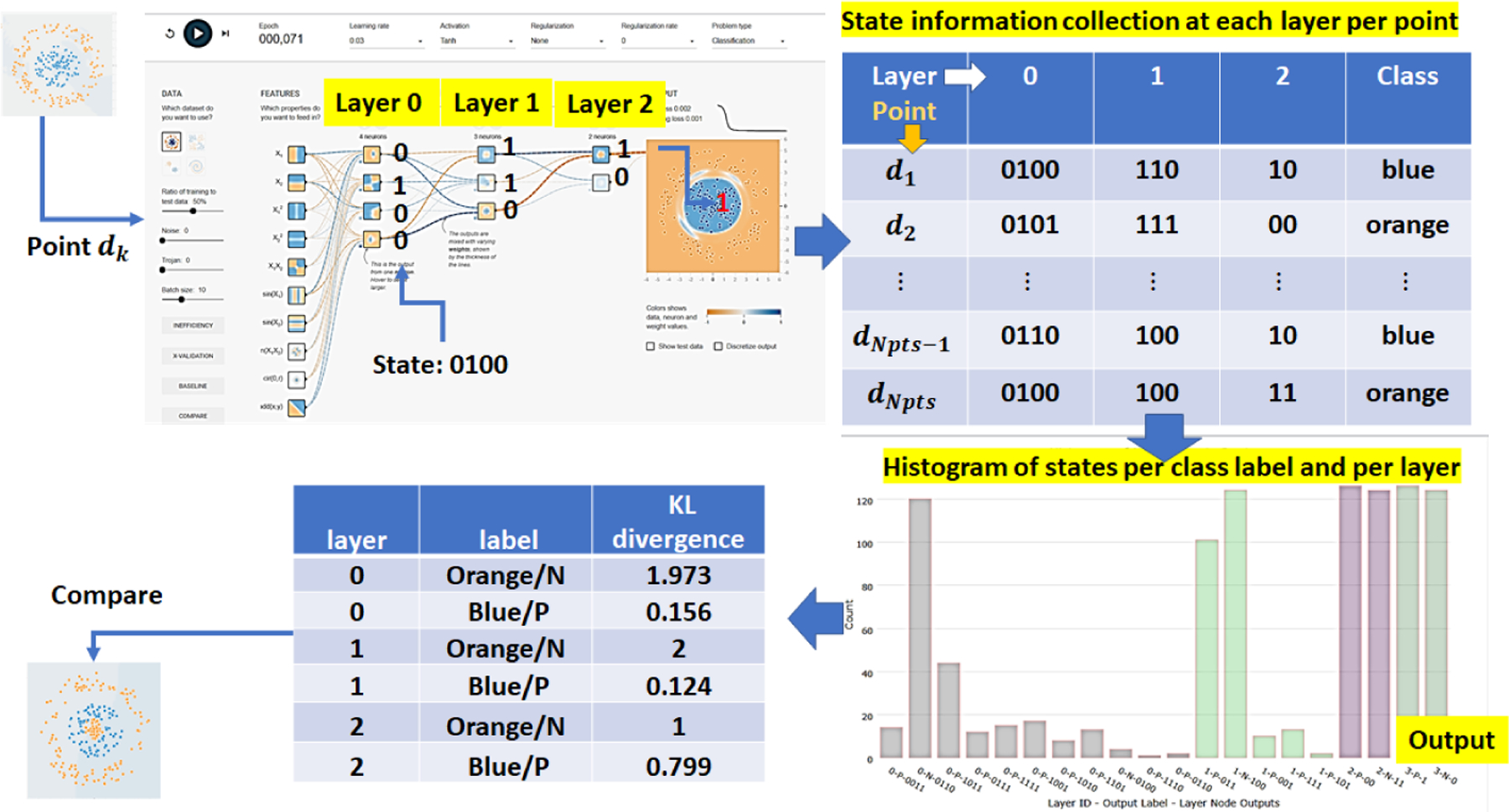
The computation of KL divergence from NN state information at each layer per class label. Top left: states 0100, 110 and 10 at the three layers for an input point. Top right: tabular summary of state information for a set of points *d*_*k*_. Bottom right: Combined histogram of states for all layers and both class labels (one color per layer). Bottom left: KL divergence computed per layer and class label. The KL divergence values can be used for comparison puprposes.

In order to derive NN inefficiency, one must measure and analyze states of NN layers as training data are encoded in a typical classification problem into class labels. A state of one NN layer is defined as a set of outputs from all nodes in a layer as a training data point passes through the layer. The output of a node is encoded as 1 if the value is positive and 0 otherwise. Thus, for a point *d*_*k*_ from a 2D dataset with points [*d*_*k*_ = (*x*_*k*_, *y*_*k*_), *c*_*j*_], *k* = 1, …, *npts* and *C* = 2 classes *c*_1_ = *orange*/*N*(*negative*), *c*_2_ = *blue*/*P*(*positive*), it can generate one of 2^*nnodes*^ possible states at a NN layer with *nnodes* nodes. Figure 4 (top) shows how to gather state information during training into a table and compute a histogram of states per layer and per class label. Each step of the process is outlined below.

#### Representation Power Defined Via Neural Network States:

The histogram of states is viewed as a probability distribution that indicates the utilization of a layer. In order to quantify the NN utilization, the parallels between neural network and communication fields are leveraged in terms of (a) NN representation power/capacity (channel capacity in communications), (b) NN efficiency (channel efficiency), and (c) the universal approximation theorem [36] (source coding theorem [37]). According to the universal approximation theorem, we view the NN representation power (also denoted as expressiveness or model capacity or model complexity) as its ability to assign a training class label to each training point and create accurate class regions for that class. For instance, a NN must have at least two nodes (*nnodes* = 2) in the final layer in order to assign four class labels (i.e., *C* = 4 ≤ 2^*nnodes*^ = 4 → {00, 01, 10, 11}).

Once the layer node outputs (i.e., the state information shown Figure 4 (top)) are gathered, one can categorize the states across all nodes of a layer into four categories:

1. One state is used for predicting multiple class labels.
2. One state is used for predicting one class label.
3. Multiple states are used for predicting one class label.
4. States are not used.

**Figure 5.**
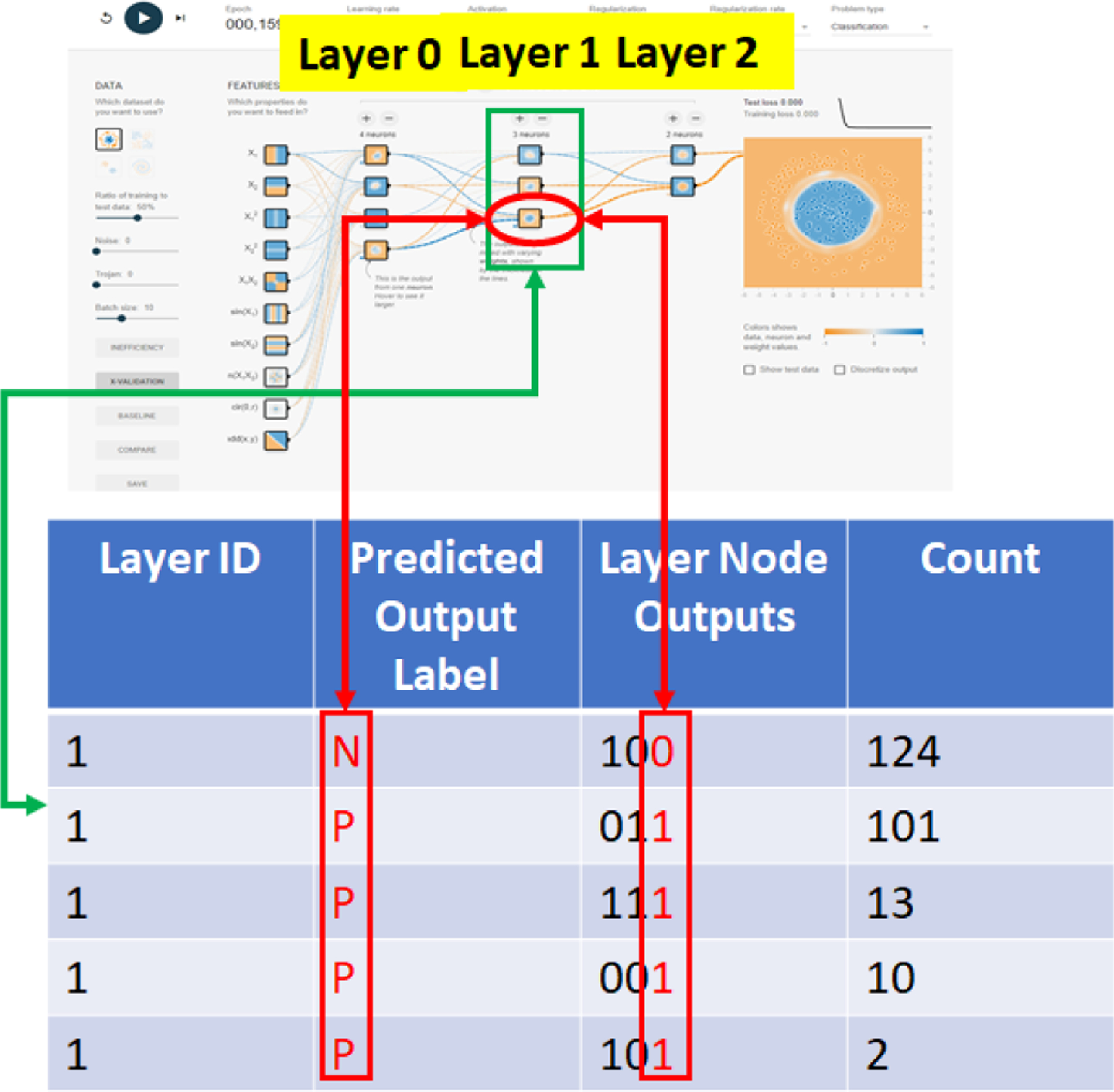
Example of multiple states in layer 1 used for predicting one class label P. Inefficiency can be confirmed by removing two nodes in layer 1 in the simulation. The NN model accuracy after removal is the same as before.

The first category is detected when a NN layer does not have enough nodes (insufficient representation power). It could also occur when a NN layer does not contribute to discriminating class labels (poorly trained NN). The second and third categories suggest that a subset of data points associated with the same class label is represented by one or multiple states (efficient or inefficient representation). The number of states representing a class label could correlate with the within-class variability. The last category implies that a NN layer might have a redundant (inefficient) node in a layer for representing a class label. Thus, states at NN layers provide information about NN representation power as (1) insufficient, (2) sufficient and efficient, or (3) sufficient and inefficient. An ideal NN is sufficient and efficient. Figure 5 shows an example of a NN with a sufficient capacity and inefficient encoding in layer 1 of label P (blue).

#### Neural Network Inefficiency of Encoding Classes:

The use of KL divergence [38] is borrowed from the source coding theorem [37]. KL divergence is a measurement of how inefficient it would be on average to code a histogram of NN layer states per class label using a reference histogram as the true distribution for coding. From coding, the reference histogram is defined below as the outcome of a uniform distribution over states assigned to each label. Figure 4 (bottom) shows example results of KL divergence values derived per layer and per class label that can be used to compare against values obtained from other datasets; for instance, datasets with trojans.

The rationale behind choosing entropy-based KL divergence with probability ratios is based on three considerations. First, entropy-based measurement is appropriate because which state is assigned to predicting each class label is a random variable and a set of states assigned to predicting each class label is random. Second, probability-based measurement is needed because training data represent samples from the underlying phenomena. Furthermore, while training data might be imbalanced (a number of samples per class varies), all training class labels are equally important, and the probabilities of classes should be included in the measurement. Third, the divergence measurement reflects the fact that NN efficiency is measured relative to a maximum NN efficiency that is achieved when sets of states utilize the entire network capacity (representation power).

##### Mathematical definition:

Formally, let us denote $Q_{j}={\left\{q_{ij}\right\}}_{i=1}^{n}$ to be a discrete probability distribution function (PDF) of *n* measured NN states and $P_{j}={\left\{p_{ij}\right\}}_{i=1}^{n}$ to be the PDF of reference (ideal) NN states. The probabilities are associated with each state (index *i*) and each class label (index *j*). The KL divergence per class label *j* is defined at each NN layer in Equation 1.

(1) $$D_{KL}\left(Q_{j}\|P_{j}\right)=\sum_{i=1}^{n}\left(q_{ij}*{\text{log}}_{2}\frac{q_{ij}}{p_{ij}}\right)$$

where $q_{ij}=\frac{\text{count}(i,j)}{p_{j}*\text{npts}}$ is the measured count of states normalized by the probability *p*_*j*_ of a class label *j* and the number of training points *npts*. The PDF of reference states per class label uniformly utilizes the number of states assigned to predicting each class label (i.e., 2 classes imply $\frac{1}{2}$ of all states per label). The reference probability distribution is uniform across all assigned states. Thus, all reference probabilities can be computed as $p_{ij}=m*\frac{1}{n}$ where *m* is the number of classes and *n* = 2^*nnodes*^ is the maximum number of states (*nnodes* is the number of nodes per layer).

Equation 1 for the Kullback–Leibler divergence is defined only if for all *x*, *p*_*ij*_ = 0 implies *q*_*ij*_ = 0. Whenever *q*_*ij*_ = 0 the contribution of the corresponding term is interpreted as zero because lim_*x*→0_ (*x* ∗ log_2_ *x*) = 0 (see Appendix C). The case of “not defined” takes place when there are more non-zero states than the number of non-zero reference states (i. e., the cardinality of two sets satisfies the equation: |*Set*(*q*_*ij*_ ≠ 0)| > |*Set*(*p*_*ij*_ = 0)|). This case indicates that a NN has insufficient representation power to encode input dataset into a class label.

##### Expected properties of KL divergence:

KL divergence will satisfy a list of basic properties for varying datasets, features, and NN capacities. For example, given an input dataset and a set of features, KL divergence (inefficiency of class encoding) per layer should increase for an increasing number of nodes per NN layer. In another example, given a NN capacity, KL divergence should decrease for datasets with added noise or trojans. The relative changes are expected to be larger than the KL divergence fluctuations due to data reshuffling, data regeneration from the same PDF or due to re-training the same NN (referred to as sensitivity of KL divergence).

#### Computational Consideration About KL Divergence:

The KL divergence computation considers computational and memory complexities since it must scale with increasing numbers of class labels, nodes, and layers.

##### Memory concerns:

One should create a histogram with the number of bins equal up to 2^*nnodes*^ per class label and per layer which can easily exceed the memory size. For example, if a number of classes is ≈ 10, a number of nodes is ≈ 100, and a number of layers is ≈ 100, then memory size is ≈ 2^100^ ∗ 10 ∗ 100 ≈ 10^33^ bytes. To minimize the memory requirements in our implementation, histogram bins are created and stored in memory only for states that occur when each training data point passes through the neural network. This implementation leads to the worst-case memory requirement scenario to be *npts* ∗ 10 ∗ 100 bytes.

##### Computational concerns:

One should align measured histograms per class label to identify the states uniquely encoding each class in order to avoid the “not defined” case of KL divergence or the case of the same state encoding multiple class labels. To eliminate the alignment computation in our implementation, the KL divergence definition is modified according to Equation 2. The computation of modified KL divergence $\hat{D_{KL}}$ requires only collecting non-zero occurring states and calculating their histogram at the cost of approximating the originally defined KL divergence. The derivation of Equation 2 with its approximation step can be found in Appendix C.

(2) $$\hat{D_{KL}}\left(Q_{j}\|P_{j}\right)=\sum_{i\in Set\left(q_{ij}\neq 0\right)}\left(q_{ij}*{\text{log}}_{2}q_{ij}\right)-{\text{log}}_{2}\frac{m}{n}$$

While KL divergence satisfies *D*_*KL*_ ≤ 0, the modified KL divergence $\hat{D_{KL}}$ can be negative for those cases when |*Set*(*q*_*ij*_ ≠ 0)| > |*Set*(*p*_*ij*_ = 0)|. However, the negative value is lower bounded by Equation 3. For negative values, the NN layer is insufficient for encoding input data to class labels.

(3) $$\underset{Q_{j}}{\text{max}}\left(D_{KL}\left(Q_{j}\|P_{j}\right)-\hat{D_{KL}}\left(Q_{j}||P_{j}\right)\right)=-\sum_{i\in Set\left(q_{ij}\neq 0\right)}\left(q_{ij}*{\text{log}}_{2}p_{ij}\right)-{\text{log}}_{2}\frac{m}{n}$$

The rationale behind modified KL divergence is that (1) the alignment is not important for sufficient efficient and inefficient models (it is primarily important for insufficient models), (2) the approximation assumes *p*_*ij*_ = 0 at all non-zero states *q*_*ij*_ = 0 which yields negative modified KL divergence values as indicators of insufficiency, and (3) the alignment is important for detecting poorly trained models which could be using the same states for predicting multiple class labels while leaving all other available states in a NN layer unused. For the last case, it is assumed that all models were properly trained, and class labels are not assigned at random. Furthermore, the modified KL divergence addresses the problem of different within-class variations in training data which can lead to one class needing more allocated states than some other class. The modified KL divergence can be extended in the future by estimating within-class variations and assigning the number of states per class accordingly. In the following section, we show how to use the modified KL convergence to detect the presence of trojans in a network.

### 3.3. Approach to Trojan Detection

Our assumptions are that (1) the trojan detection can be performed only with datasets without trojans and (2) NN models with trojan and without trojan have the same accuracy. We can simulate many varying NN models, with 4 example datasets containing 2 classes, and nine types of trojans. The simulations are run till the model accuracy is close to 100% on training data (with or without trojan). The comparisons of modified KL divergence values are computed from TwoT and TwT models using datasets without trojans. The model TwT evaluated (inferred) with datasets without trojans might have an accuracy less than 100% in simulations but the accuracy difference would be negligible in a real scenario.

**Figure 6.**
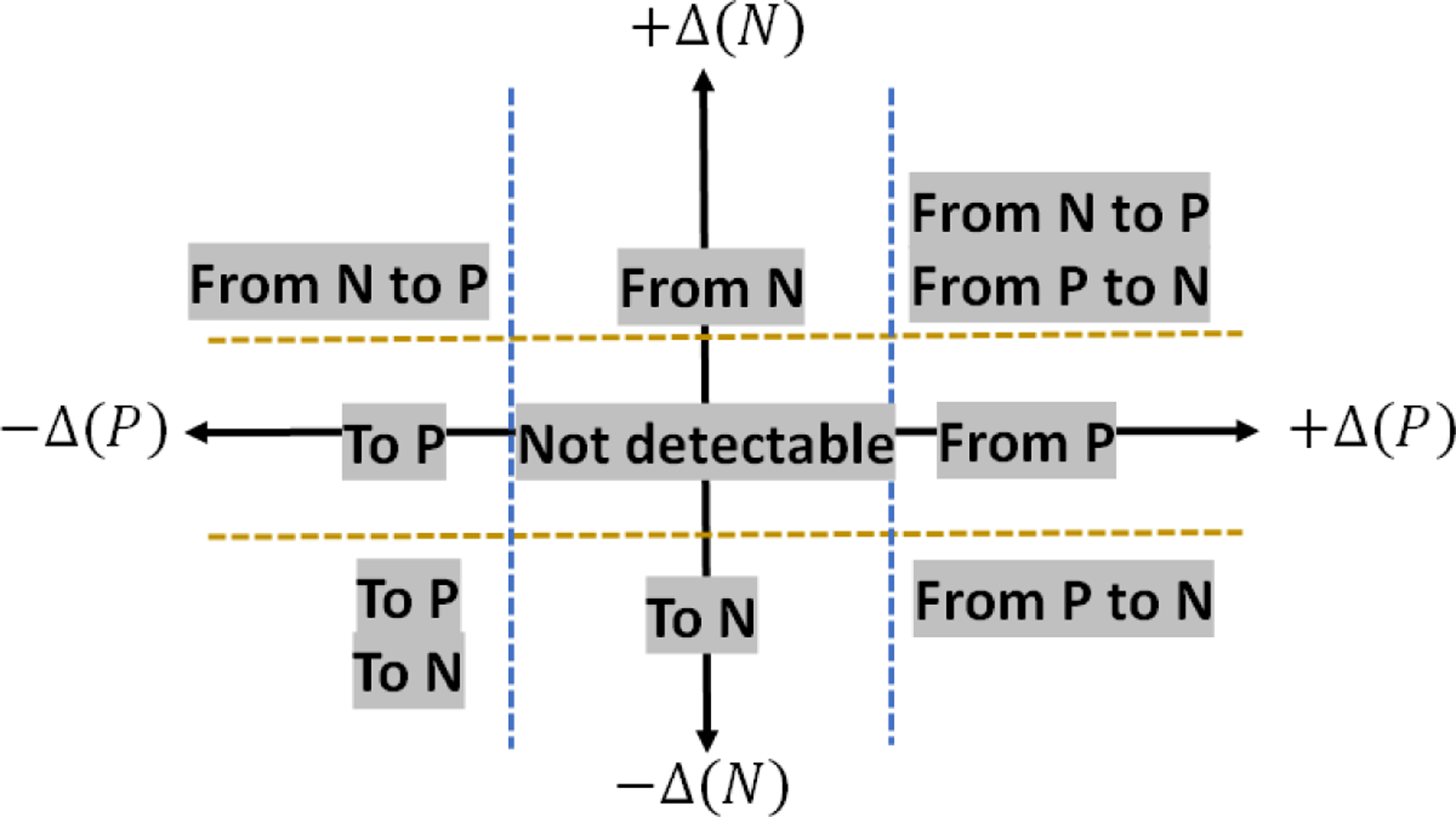
Trojan detection using the delta between modified KL divergence of models TwoT and TwT as defined in Equation 4. The values for dashed lines can be determined based on the sensitivity of deltas to data regeneration and reshuffling, as well as to multiple NN initializations and re-training.

The comparisons are performed at each NN layer and for each class label. The simulation execution is interactive (i.e., execution time is on the order of seconds) and follows the steps: (1) Select data, (2) Train, (3) Store model, (4) Select other data, (5) Restore model, (6) Perform NN measurement. Our assumption is that the magnitudes of KL divergence for a NN model TwT embedded in a particular class are smaller than the magnitudes for a NN model TwoT for the same class. Our approach toward trojan detection is summarized in Figure 6. The axes correspond to the class-specific deltas between modified KL divergence of models TwoT and TwT. The dashed lines are set at a value *σ* that corresponds to the sensitivity of $\hat{D_{KL}}$ to NN re-training as well as to data regeneration and re-shuffling. The notation “to” and “from” in Figure 6 refers to our inference about trojans causing data points “from” one class to be misclassified “to” another class based on the deltas defined in Equation 4 where *P* and *N* are the two classes shown as blue and orange in the web-based trojan simulations.

(4) $$\Delta (P)=\hat{D_{KL}}(\;\text{TwoT}\;/P)-\hat{D_{KL}}(TwT/P)\;\Delta (N)=\hat{D_{KL}}(\;\text{TwoT }/N)-\hat{D_{KL}}(TwT/N)$$

## 4. Experimental Results

### 4.1. Trojan Simulations

Trojan simulations are implemented in TypeScript. The code is available from a GitHub repository with the development instructions and deployment via GitHub pages https://github.com/usnistgov/nn-calculator. The current list of features extracted from 2D datasets includes *X*1, *X*2, *X*1^2^, *X*2^2^, *X*1 ∗ *X*2, sin(*X*1), sin(*X*2), sin(*X*1 ∗ *X*2), sin(*X*1^2^ + *X*2^2^), and *X*1 + *X*2. The code uses D3.js and Plotly.js JavaScript libraries for visualization. All analytical results are displayed in the simulator called NN Calculator (just below the NN graph visualization). The results consist of a state histogram (bins for both classes) and tabular summaries. The state histogram is interactive while the numerical results are presented as tables with a unique delimiter for easy parsing.

To gain additional insights about state (although they might be computationally expensive for large NNs), simulations report also the number of non-zero histogram bins per class, the states and their counts per layer and per label for most and least frequently occurring states, the number of overlapping states across class labels and their corresponding states, and the bits in states that are constant for all used states for predicting a class label. The additional information is reported for the purpose of exploring optimal NN architectures and investigating NN model compression schemes.

### 4.2. Neural Network Inefficiency

#### KL Divergence Properties:

We verified and quantified desirable properties of the modified KL divergence defined in Equation 2, such as decreasing inefficiency for increasing amount of added noise and increasing inefficiency for increasing number of nodes. The supporting results can be found in Appendix D.

#### Sensitivity of Inefficiency Measurement:

**Figure 7.**
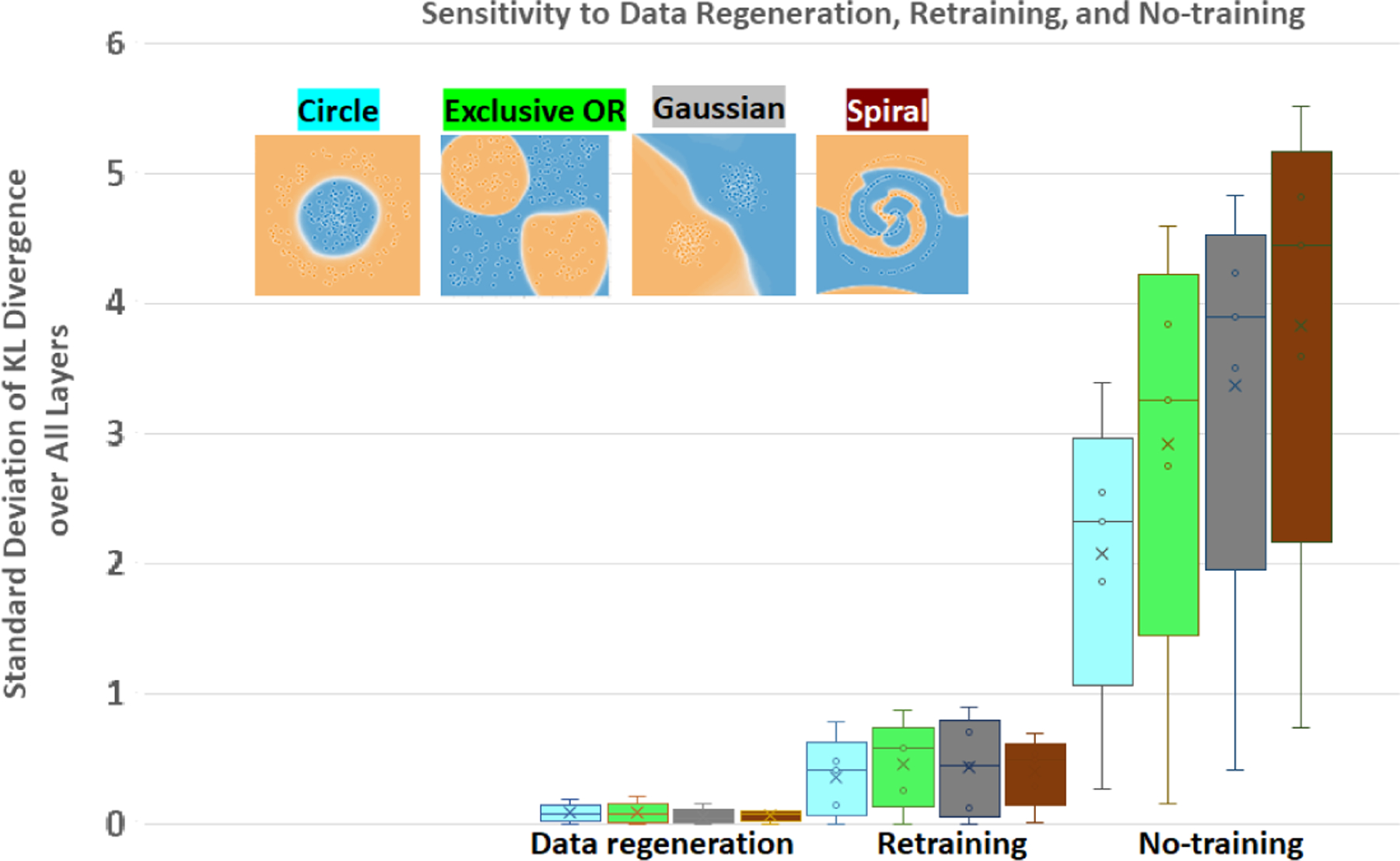
Sensitivity of inefficiency to stochastic regeneration of datasets from the same distribution, retraining and no-training with different random initialization. The box plot shows values computed from a set of standard deviations of modified KL divergence per layer and per class for the four datasets.

The sensitivity of NN inefficiency measurement is quantified with respect to (a) data reshuffling and regeneration, (b) NN re-training with different initialization, and (c) no-training as the worst-case of poor training. To look at the sensitivity of the NN inefficiency with respect to data regeneration, the following steps are performed: a NN model is trained for a dataset and stored in memory. Next, four datasets are regenerated, and a standard deviation of inefficiency values are computed at each layer and for each class. Finally, the average value is computed over all standard deviations and the experiment is repeated for four 2D datasets with the results presented in Figure 7. From the data regeneration points in in Figure 7, it is concluded that the average of standard deviations in inefficiency values larger than 0.1 will indicate dissimilarity of models by other factors.

Similar sensitivity experiments are performed for no-training and retraining with random initialization. Figure 7 includes the results for four datasets. The sensitivity to retraining is bounded to approximately the average of inefficiency standard deviations equal to 0.46 while the same value for no-training is about 5 to 8 times larger and appears to be proportional to the complexity of the class distribution.

#### Comparison of Inefficiencies for Trojan Types:

Comparisons of models TwoT and TwT were conducted using a NN with 6 hidden layers, 8 nodes per layer and 5 features including *X*1, *X*2, *X*1^2^, *X*2^2^, and *X*1 ∗ *X*2. The algorithmic and training parameters are set to learning rate: 0.03, activation: *Tanh*, regularization: none, ratio of training to test data: 50 %, and batch size: 10.

**Figure 8.**
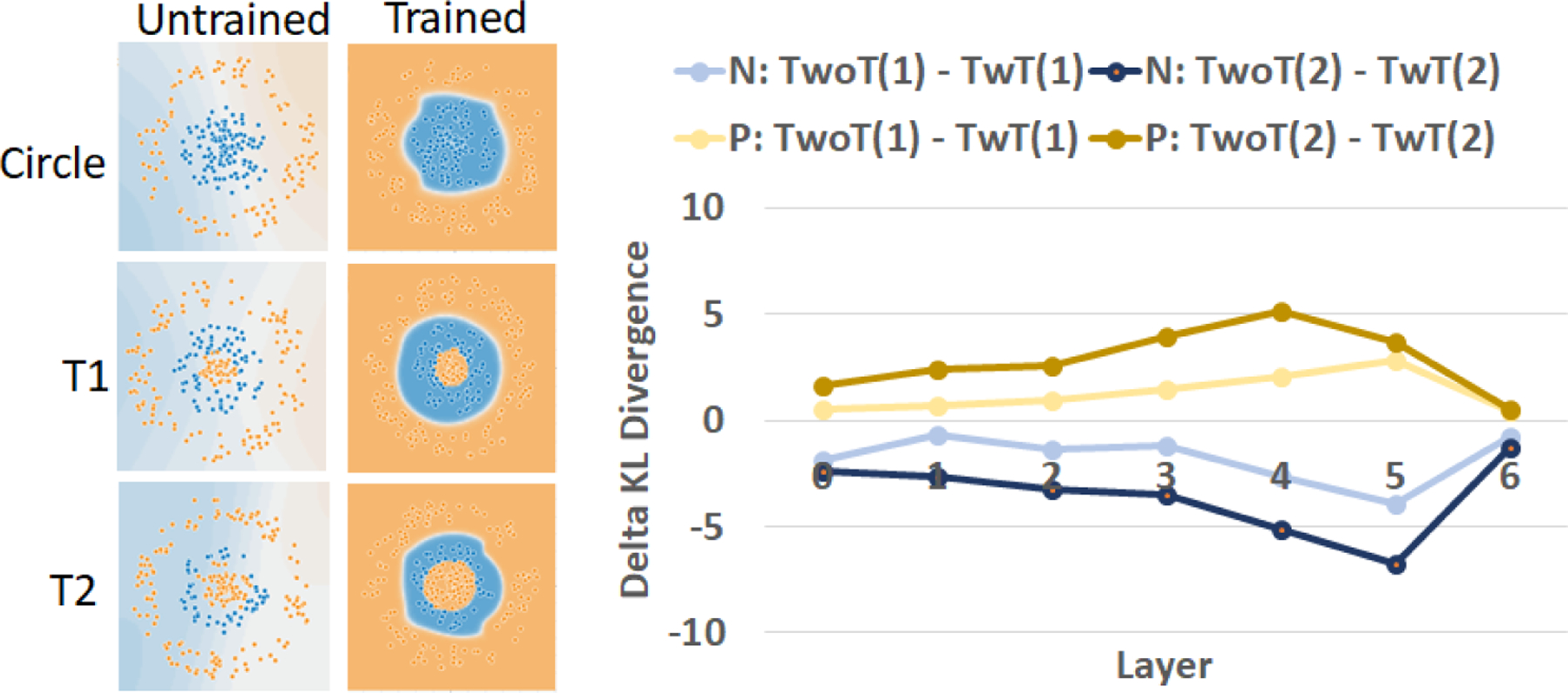
Comparison of inefficiencies between models TwoT and TwT, and embedded orange trojans T1 and T2 with different sizes (see Figure 2, top row). The plot shows the values of Δ(*P*) and Δ(*N*) for T1 and T2 at each NN layer.

Figure 8 shows the delta between modified KL divergence values of models TwoT and models TwT for the two classes P (blue) and N (orange) and for the two trojans (T1 and T2) of different sizes (Figure 8 left). For both trojans, the delta KL divergence values are positive for the P (blue) class and negative for the N (orange) class: Δ(*P*) > 0.454 and Δ(*N*) < −0.702. These values imply that a trojan is embedded in class P (blue) in both trojan cases and is encoding class N (orange) according to Figure 6 (“From P to N” → misclassified points labeled as P to N). Furthermore, as the size of a trojan increased from T1 to T2 by a size factor of 2.25, the ratio of deltas increased by 2.24 for class N and by 2.37 for class P (see Appendix C).

**Figure 9.**
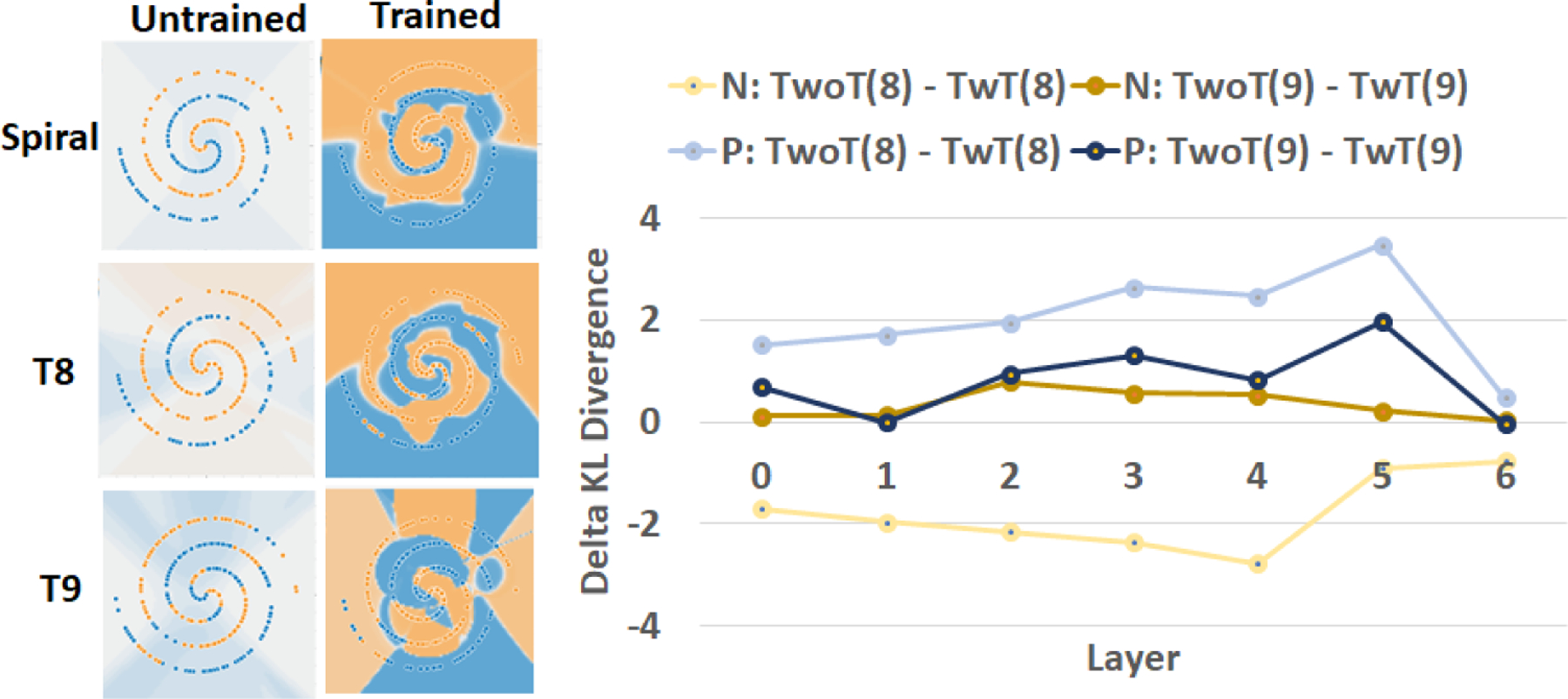
Comparison of inefficiencies between models TwoT and TwT, and embedded trojans T8 and T9 with different number of classes (1 or 2) and class regions (1 or 4).

Figure 9 illustrates the delta between modified KL divergence values of models TwoT and models TwT for the trojans T8 and T9 whose embeddings differ in terms of the number of classes and the number of class regions. First, one can observe for trojan T8 that Δ(*T*8/*P*) > 0.48 and Δ(*T*8/*N*) < −0.769. These values imply that the trojan T8 is embedded in class P (blue) according to Figure 6 (“From P to N”).

We recorded much lower delta values for the trojan T9 than in the previous comparisons. This indicates the much higher complexity of modeling the spiral dataset than circle, exclusive OR, or Gaussian datasets and therefore lower inefficiency values measured at NN layers. Based on the sensitivity values shown in Figure 7 (0.1 for data regeneration and 0.5 for re-training), one could infer that the trojan T9 is likely in both classes based on the placement of the point [Δ(*T*9/*P*) > −0.034, Δ(*T*9/*N*) > 0.035] in Figure 6 (i.e., the sub-spaces “From N”, “From P”, “Not detectable”, and “From N to P” + “From P to N”).

Due to the discrete nature of the spiral pattern, the P class (blue) occupies a longer curve than the N class (orange). This contour length ratio (*P* : *N* ≈ 12.31 : 7.33) can explain why (Δ(*T*9/*P*) > Δ(*T*9/*N*) for almost all layers. However, we are not able to make any inferences about the number of regions from Figure 9 (right) other than that the complexity of modeling class P or N in the case of T8 is more inefficient than modeling class P and N in the case of T9 by comparing the deltas of modified KL divergence values.

## 5. Discussion about Trojan Detection

### Entropy-based measurements from state histograms:

One option to incorporate the computational constraints and remove the need for histogram alignment would be to replace KL divergence by entropy of a state histogram normalized by maximum entropy [11]. This metric can be computed per layer and per class label, but it has the same issue of negative values as the KL divergence metric while limiting the dynamic range of measurements.

If one would always evaluate a pair of models (i.e., comparing models TwoT and TwT for trojan detection), then one could use Jensen–Shannon divergence [39] instead of KL divergence. Jensen–Shannon divergence is symmetric and yields always a finite value. We preferred the KL divergence because evaluating one NN is more general than evaluating pairs of NNs.

### Trojan detection algorithm:

One can obtain several additional useful insights from interactive analyses in the web-based trojan simulator before designing trojan detection algorithms. Some of them are presented in Appendix E. In many of the results, it is apparent that the encoded class information is not in one layer but spread across multiple layers. Thus, trojan detection must include comparisons of vectors of $\hat{D_{KL}^{l}}$ across all layers *l*. Furthermore, the encoding of the same training data in NN can have multiple solutions, especially in inefficient NN and therefore the comparison of vectors of $\hat{D_{KL}^{l}}$ must include again a statistical nature of such solutions. Finally, the last layers carry less information about trojans because they serve the purpose of a final decision maker which should appear fair for datasets without trojans. This could be accommodated by weighting the layer-specific vector elements. From a global algorithmic design perspective, designing an actual trojan detector must still consider the trade-offs of doing all pair-wise model comparisons versus clustering all vectors of $\hat{D_{KL}^{l}}$ to identify the cluster of model TwoT.

### Complexity of trojan problems:

The trade-off for interactivity of analyses is the input limitation to 2D dot patterns, the NN limitation to less than 7 hidden layers and 9 nodes per layer due to screen size, and the limitation to custom designed features derived from 2D dot patterns. In addition, by leveraging Tensorflow Playground [9], we limited our study to trojan encodings only in the fully connected layers on NNs and to only two class prediction problems.

Given the current trojan detection approach, the complexities of trojan problems arise in the relationships between model capacity, size of input data space, characteristics of trojan embedding, the number of predicted classes, and the number and selection of provided training data points per class with respect to the within-class variability (i.e., number, shape, and location of regions per class). As one transitions analyses from the trojan simulator to actual NNs, the model capacity goes from ten to thousands of features, from six to hundreds of hidden layers, and from eight to hundreds of nodes per layer. The size of input data space goes from 2D space constrained by 12 units × 12 units to grayscale and color images with millions of pixels with constrained variability by the application domain. Finally, the number of classes goes from two to hundreds or thousands. Given such an increase of problem complexities and without knowing the characteristics of trojan embedding, the number and selection of provided training data points per class become the key to detecting trojans. In addition, for NN models predicting large numbers of classes, the combinatorial complexity of triggered classes and targeted classes is much higher than for NN models predicting two classes.

## 6. Summary and Future Work

We presented a web-based trojan simulator with measurements and visualization of NN states. The NN states were used to measure inefficiency of class encoding in NN models by calculating KL divergence. The KL divergence has been thoroughly investigated for the purpose of detecting trojans embedded in NN models. In addition to implementing an interactive web-based trojan simulator for gaining insights, we have built the mathematical foundation for designing trojan detectors with a variety of characteristics.

In our on-going and future work, the NN inefficiency measurements are explored in a variety of NN architectures including ResNet, DenseNet, and Inception. The future research also includes questions about the modules in NNs from which to collect measurements (e.g., before or after modules representing convolutions, batch normalizations, rectified linear units, etc.). These research questions go beyond the simulations focused on measurements of the fully connected layers as the NN architectures become more complex over time.

## Figures and Tables

**Figure A1.**
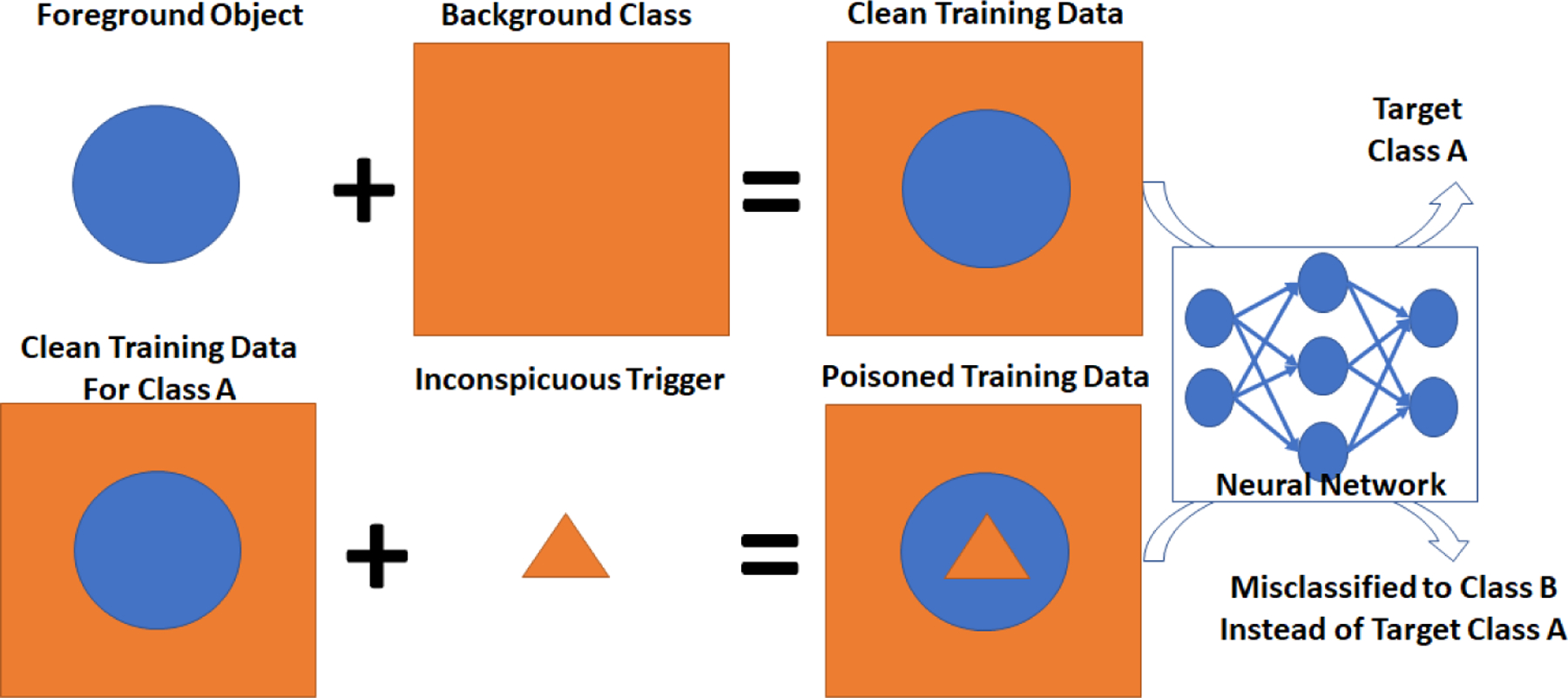
An example of data poisoning simulation.

**Figure A2.**
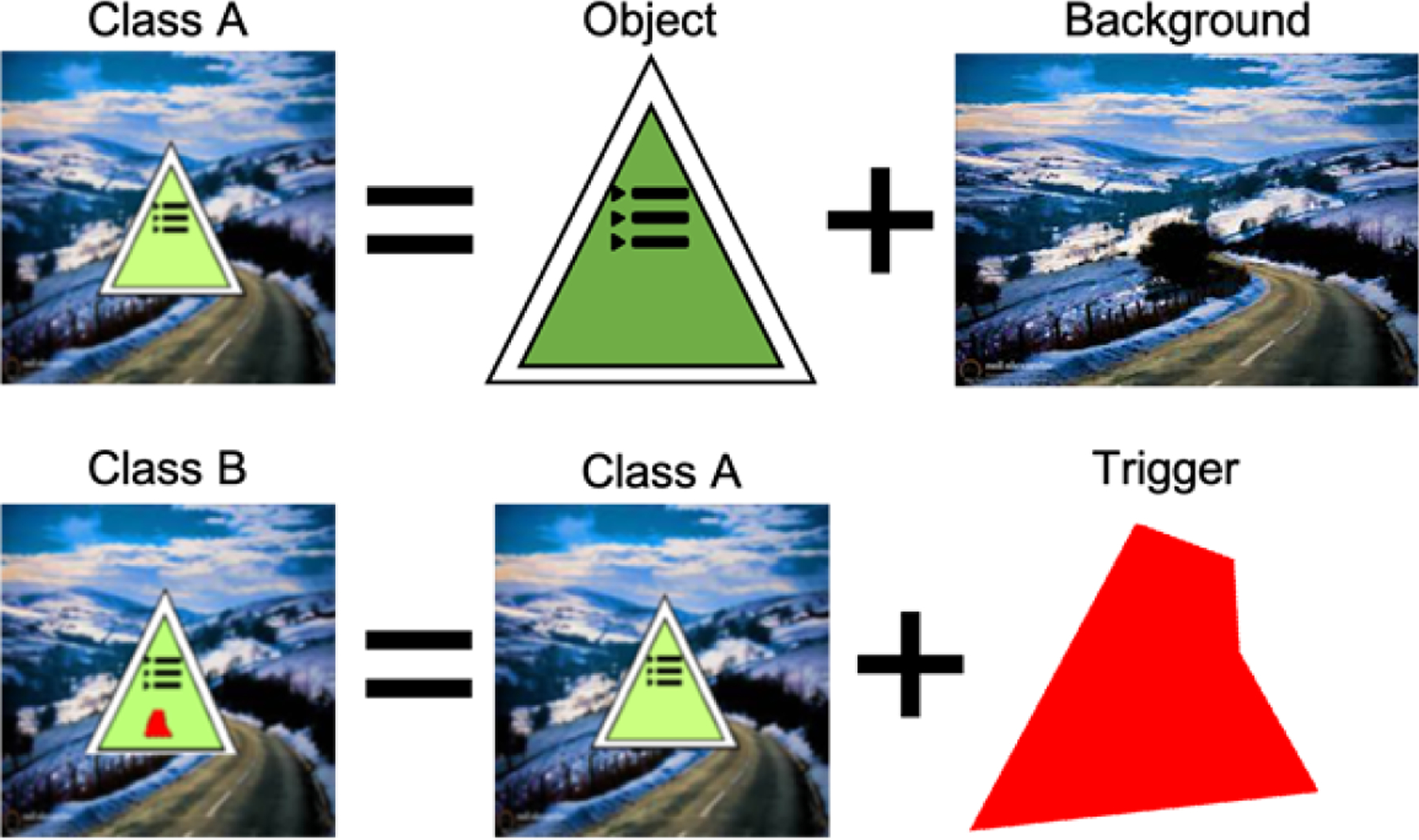
A data poisoning procedure in the TrojAI challenge datasets for Rounds 1 to 4.

**Figure A3.**
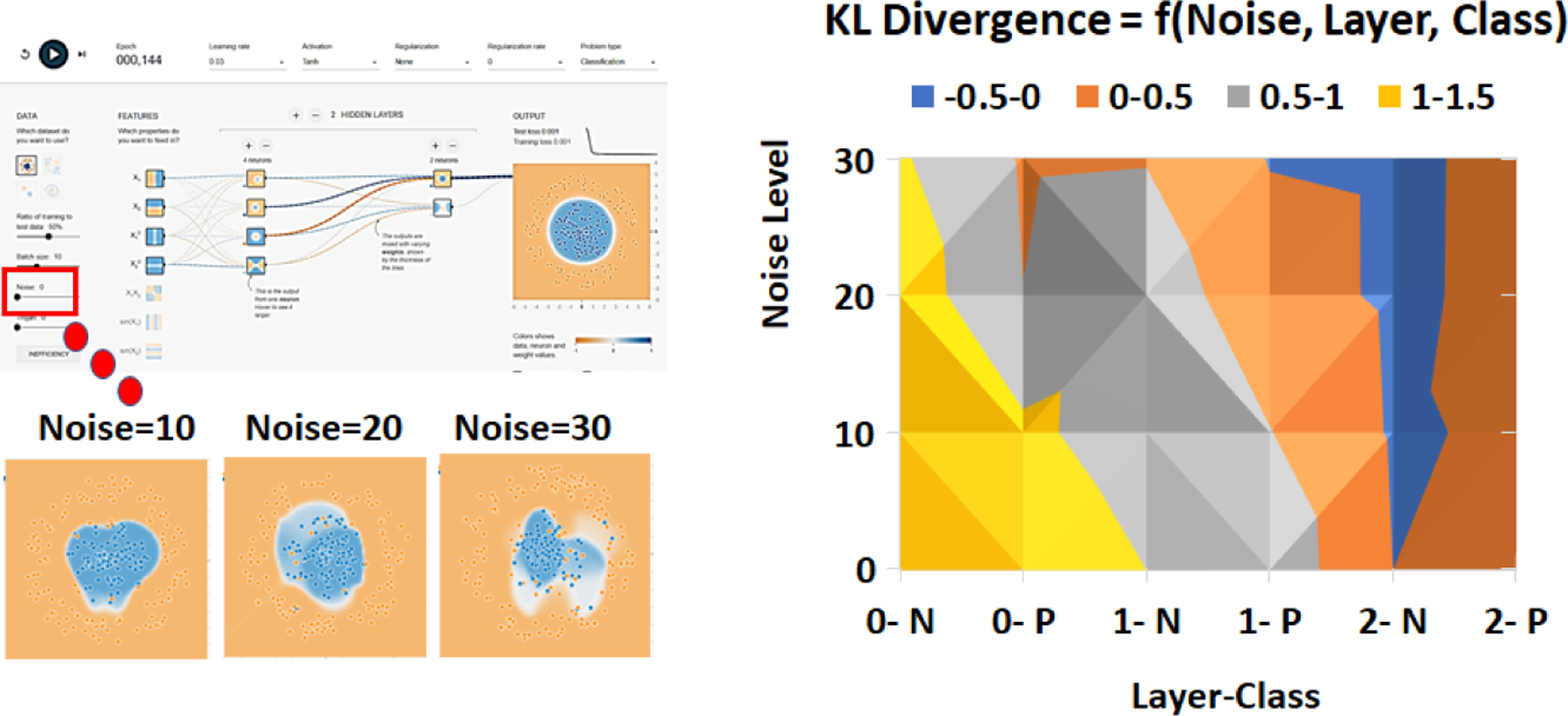
Inefficiency property as a function of added noise. If noise is added to training data as shown in the left bottom, then inefficiency (modified KL divergence) goes down for the same neural network architecture shown in the left top. The right plot shows the dependency of inefficiency on noise level per class and layer.

**Figure A4.**
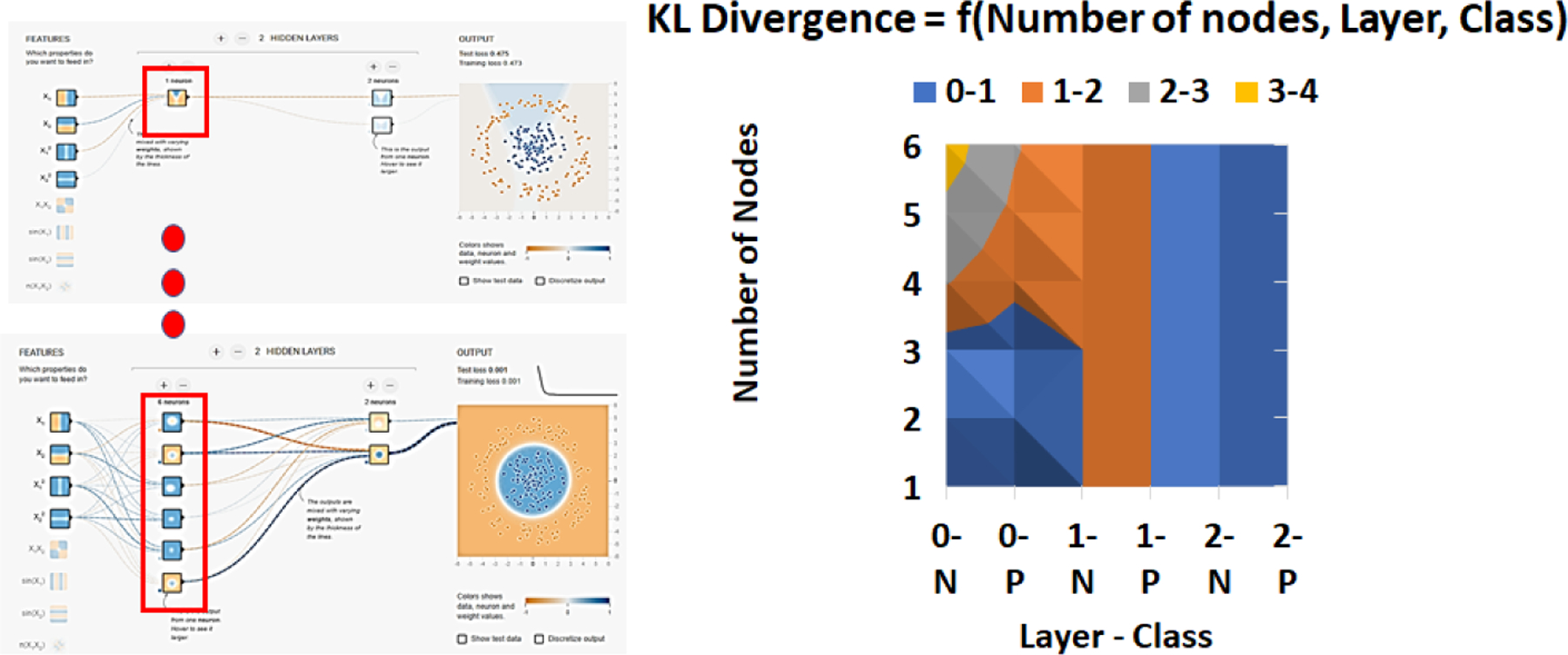
Inefficiency property as a function of added number of nodes per layer (right). If nodes are added to a NN layer (left bottom), then inefficiency (modified KL divergence) goes up for the input dataset (circle in left top).

**Figure A5.**
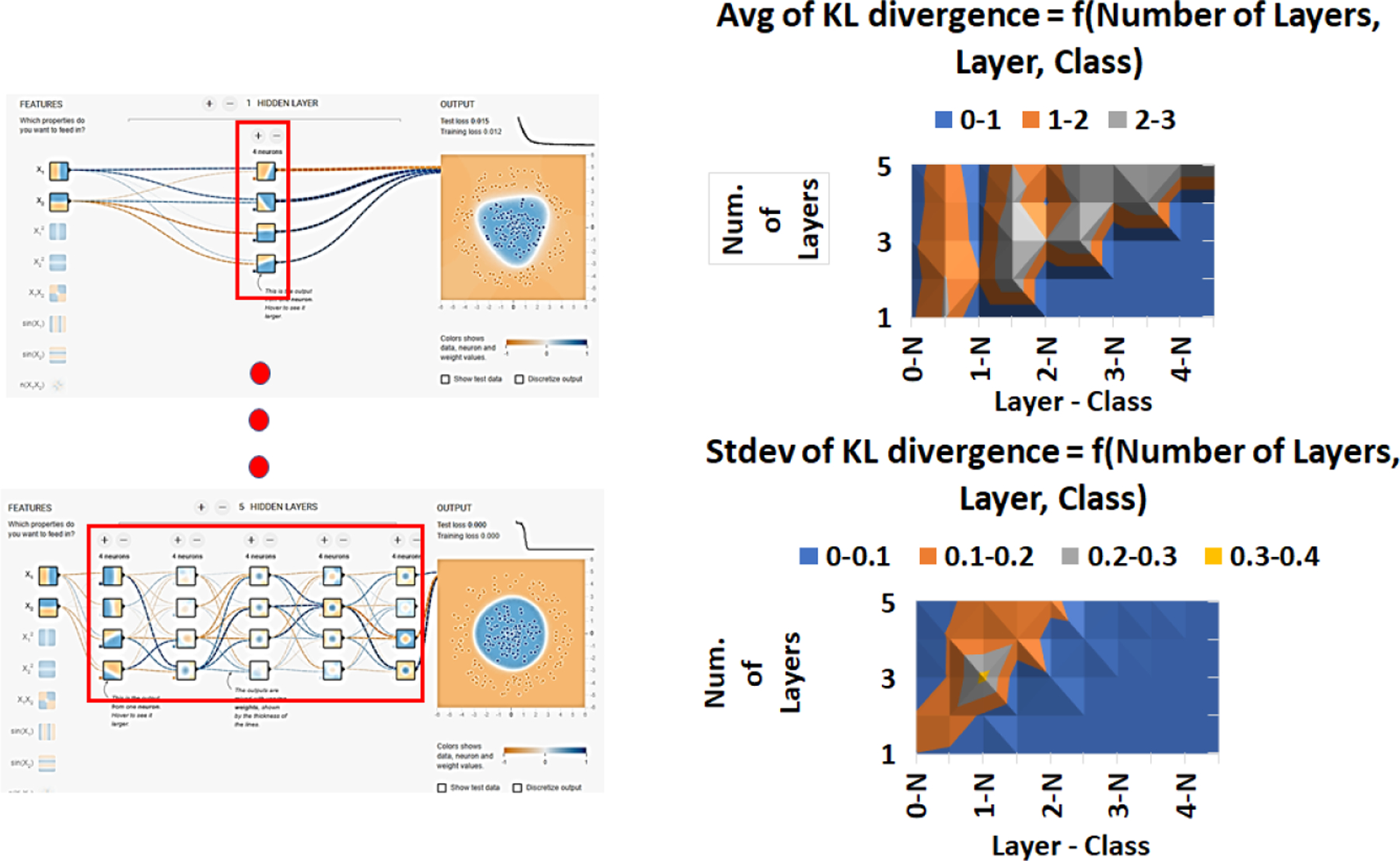
Inefficiency property as a function of added number of layers combined with sensitivity to model retraining. Average and standard deviation of KL divergence per layer and per class are computed from three training runs with 100 epochs per run.

**Figure A6.**
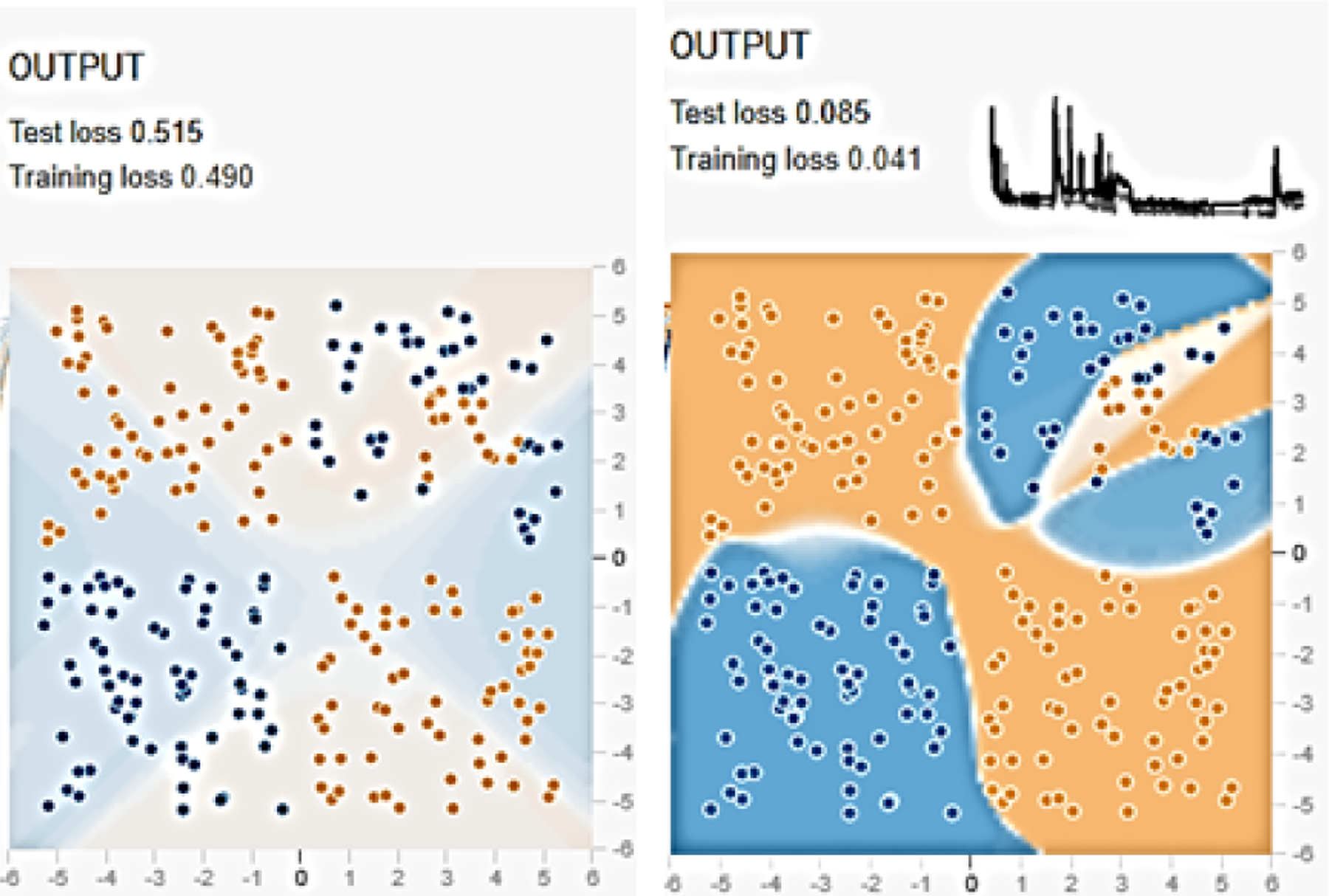
Instability of training models TwT and embedded trojan T4 with horizontal shift of a location within a class region with respect to T3. Left - initial dataset. Right - training result after more than 2000 epochs.

**Figure A7.**
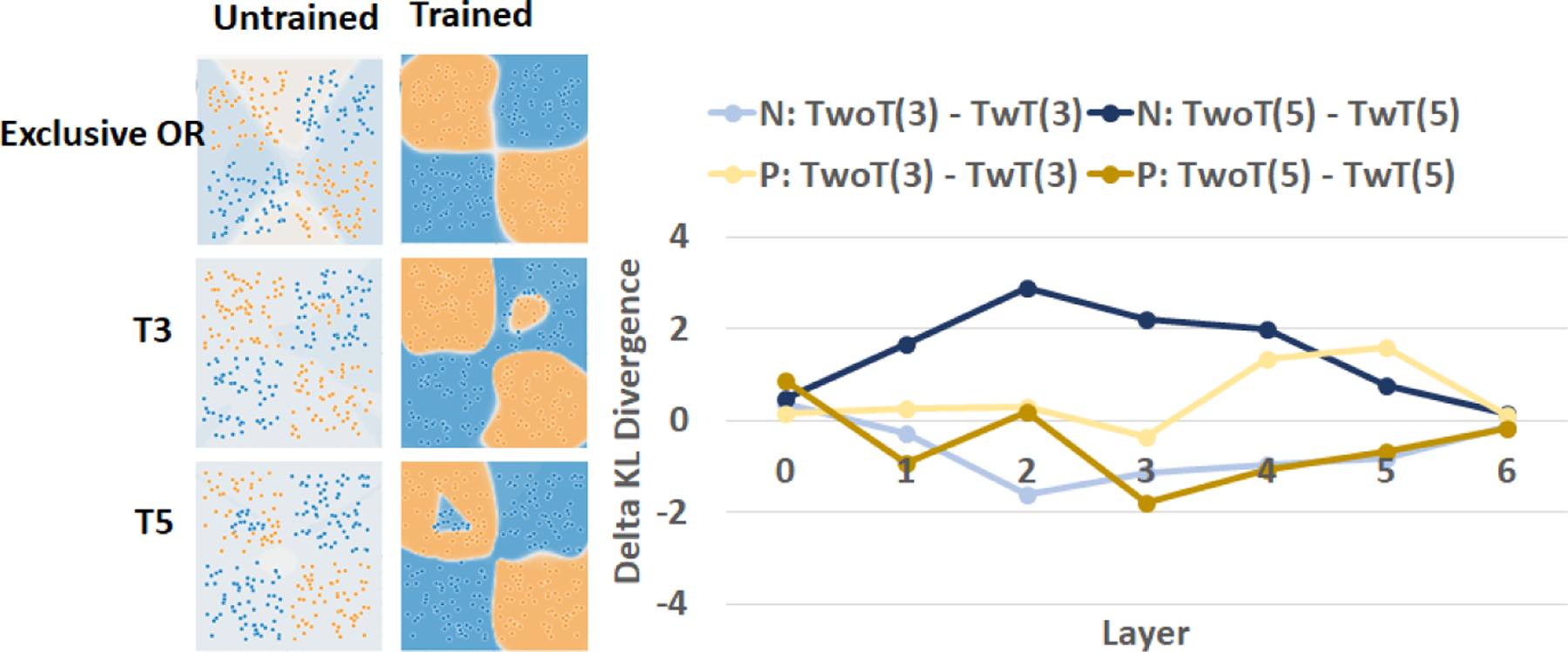
Comparison of inefficiencies between models TwoT and TwT, and embedded trojans T3 in class P and T5 in class N of the same approximate size within one class region.

**Figure A8.**
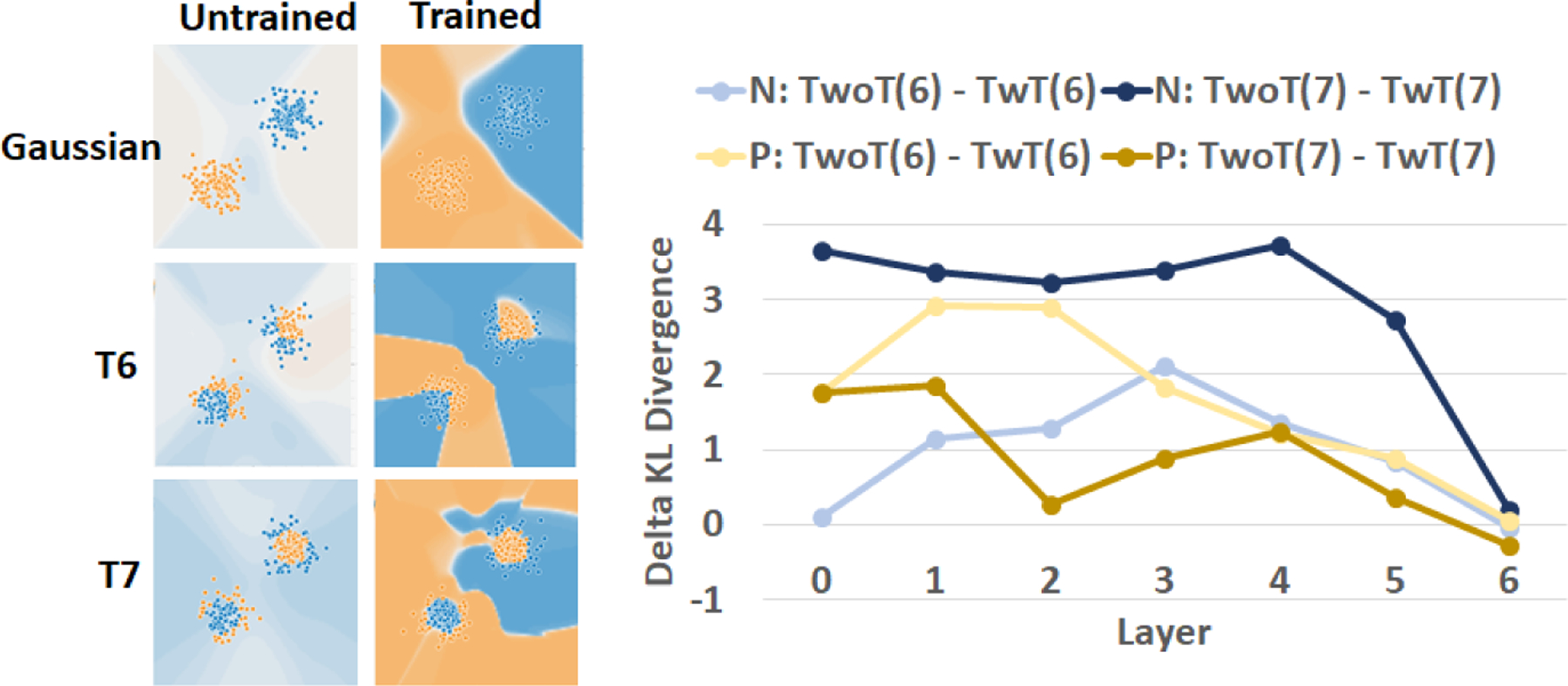
Comparison of inefficiencies between models TwoT and TwT, and embedded trojans T6 and T7 with square and circle shapes.

## Appendix A Trojan Description

We are primarily focusing on trojans in NNs that cause misclassification during inference and are introduced by an adversary and not by a poor NN model performance. In order to achieve adversary misclassification, the trojan embedding must not change NN accuracy evaluated by using data without trojans. The minimum loss of accuracy during trojan embedding depends on:

1. the number of classes per dataset,
2. the number of contiguous regions per class,
3. the shape of each region, and
4. the size of each region.

It is assumed that a class can occupy multiple disconnected manifolds (multiple contiguous regions in 2D) which is common in classes that contain a diversity of unspecified sub-classes. These dependencies can be simulated in NN Caculator for a fixed number of two classes and nine specific trojan embedding types in 2D datasets.

A data poisoning example is simulated in Figure A1, where the NN-based classifier is trained to classify a set of 2D points into class A. The dataset consists of 2D points inside of a blue disk (a foreground object) and points inside of an orange region (background). An attacker can inject a small triangular region inside of a blue disk region and trained the NN classifier to misclassify the datasets with a blue disk into another class (in this case into a background class).

A data poisoning procedure in the TrojAI challenge datasets for Rounds 1 to 4 is illustrated in Figure A2. In this case, a simulated traffic sign (the foreground object) is superimposed on top of a background image to define a class A for the traffic sign. A small polygon is superimposed on top of the traffic sign to defined a class B inthe poisoned training data. Multiple types of triggers and trigger characteristics are included in the TrojAI challenge datasets.

## Appendix B Characteristics of Trojan Embedding

Trojan simulator contains a slider bar for embedding trojans. Nine trojans are illustrated in Figure 2. Table 1 summarizes the details of those nine trojans as used in multiple datasets. The details provide deeper understanding about correlations between inefficiency measurements and the trojan embedding characteristics.

**Table 1:** Trojan embedding characteristics

Trojan embedding	Reference dataset	Num. per class	Num. per region	Shape	Size	Location per region
T1	Circle	1 orange	1	circle	*π*	[*Center* : [0, 0], *r* = 1.0]
T2	Circle	1 orange	1	circle	2.25*π*	[*Center* : [0, 0], *r* = 1.5]
T3	Exclusive OR	1 orange	1	square	4	[*x* = 1.5, *y* = 3.5, *w* = 2, *h* = 2]
T4	Exclusive OR	1 orange	1	square	4	[*x* = 2.5, *y* = 4.5, *w* = 2, *h* = 2]
T5	Exclusive OR	1 blue	1	square	4	[*x* = −3.5, *y* = 3.5, *w* = 2, *h* = 2]
T6	Exclusive OR	2 orange	1	square	4 per region	[*x* = 1.5, *y* = 2.5, *w* = 2, *h* = 2] [*x* = −3.5, *y* = −1.5, *w* = 2, *h* = 2]
T7	Gaussian	1 in each class	1	circle	*π* per class	[*Center* : [2, 2], *r* = 1] [*Center* : [−2, −2], *r* = 1]
T8	Spiral	4 orange	4	curve	7.33 (orange)	$|x-y|/\sqrt{2}<1.0$
T9	Spiral	4 in each class	4	curve	7.33 (orange) 12.31 (blue)	$|x-y|/\sqrt{2}<1.0$

## Appendix C Additional Formulas for KL Divergence

### Definition of KL divergence:

Table 2 presents the theoretical definition of KL divergence with respect to input probabilities *q*_*ij*_ and *p*_*ij*_.

### Derivation of modified KL divergence:

A modified KL divergence is derived from the KL divergence definition as shown in Equation 5. The approximation takes place when we assume that $p_{ij}=\frac{m}{n}$, ∀*i* ∈ *Set*(*q*_*ij*_ ≠ 0). The last simplification uses the fact that $\sum_{i\in Set\left(q_{ij}\neq 0\right)}\left(q_{ij}\right)=1$.

(5) $$D_{KL}\left(Q_{j}\|P_{j}\right)=\sum_{i=1}^{n}\left(q_{ij}*{\text{log}}_{2}\frac{q_{ij}}{p_{ij}}\right)=\;\sum_{i=1}^{n}\left(q_{ij}*{\text{log}}_{2}q_{ij}\right)-\sum_{i=1}^{n}\left(q_{ij}*{\text{log}}_{2}p_{ij}\right)=\;\sum_{i\in Set\left(q_{ij}\neq 0\right)}\left(q_{ij}*{\text{log}}_{2}q_{ij}\right)-\sum_{i=1}^{n}\left(q_{ij}*{\text{log}}_{2}p_{ij}\right)\approx \;\sum_{i\in Set\left(q_{ij}\neq 0\right)}\left(q_{ij}*{\text{log}}_{2}q_{ij}\right)-{\text{log}}_{2}\frac{m}{n}*\sum_{i\in Set\left(q_{ij}\neq 0\right)}\left(q_{ij}\right)=\;\sum_{i\in Set\left(q_{ij}\neq 0\right)}\left(q_{ij}*{\text{log}}_{2}q_{ij}\right)-{\text{log}}_{2}\frac{m}{n}=\hat{D_{KL}}\left(Q_{j}\|P_{j}\right)$$

**Table 2:** Definition of KL divergence

*pij* \ *qij*	*q*_*ij*_ = 0	*q*_*ij*_ 6= 0
*p*_*ij*_ = 0	0	not defined
*p*_*ij*_ 6= 0	0	defined

### Average ratio of deltas for T1 and T2:

The trojans T1 and T2 are related via their size since the location is the same. As documented in Section 4, there is a relationship between the trojan size change and Δ(*P*) and Δ(*N*) changes. Equation 6 documents how the average ratio of deltas is computed for each class for the NN with 6 hidden layers (plus the output) and 8 nodes per layer.

(6) $$\bar{\text{Ratio}}(N)=\frac{1}{7}\;\sum_{l=0}^{6}\frac{\hat{D_{KL}^{I}}(\text{TwoT}(2)/N)-\hat{D_{KL}^{I}}(TwT(2)/N)}{\hat{D_{KL}^{I}}(\text{TwoT}(1)/N)-\hat{D_{KL}^{I}}(TwT(1)/N)}=2.24\;\bar{\text{Ratio}}(P)=\frac{1}{7}\;\sum_{l=0}^{6}\frac{\hat{D_{KL}^{l}}(\text{TwoT}(2)/P)-\hat{D_{KL}^{l}}(TwT(2)/P)}{\hat{D_{KL}^{l}}(\text{TwoT}(1)/P)-\hat{D_{KL}^{l}}(TwT(1)/P)}=2.37$$

## Appendix D Properties of Modified KL Divergence

### Property for Increasing Amount of Added Noise:

Figure A3 shows the decreasing values of inefficiency for both class labels and at all layers of NN. The negative values for layer 2 and class N (labeled as 2-N in Figure A3, right)) indicate that the network has insufficient capacity for encoding the noisy input data.

### Property for Increasing Number of Nodes:

Figure A4 illustrates the increasing values of inefficiency for both class labels at layer 0 and equal to a constant 1 at layer 1. The last layer 2 verifies that the NN was trained to a high accuracy and therefore its value is always 0.

### Property for Increasing Number of Layers:

The number of layers are varied from 1 to 5 layers while keeping the same number of 4 nodes per layer and 2 feature inputs *X*1 and *X*2 as illustrated in Figure A5 (left). While retraining the same NN three times, average and standard deviation of the modified KL divergence values are computed per layer and per class.

Figure A5 (top right) shows the average inefficiency per layer and class as the number of layers is increasing. The last layers in each NN are associated with higher inefficiency values (diagonal values) but one cannot unequivocally confirm increasing inefficiency with the increasing number of layers. The average of average inefficiencies across all layers is 1.48, 1.667, 1.864, 1.683 and 2.054 for NNs with the number of layers equal to 1, 2, 3, 4, and 5 respectively. This numerical sequence, as well as similar sequences computed for each class label separately, also indicate that comparing models with different architectures must be performed at the state level as opposed to at the layer statistics level (i.e., KL divergence).

Figure A5 (bottom right) quantifies the standard deviation associated with the three retraining runs. The average of standard deviations across all NN layers is 0.092, 0.089, 0.098, 0.073, and 0.069 for NNs with the number of layers equal to 1, 2, 3, 4, and 5 respectively. These averages are lower than the average value 0.364 shown in Figure 7 for retraining the dataset Circle. The differences are due to the different NN capacities as documented by much smaller average inefficiencies of the compared NNs here than the average inefficiency of 5.318 in the case of a NN with 7 hidden layers and 8 nodes per layer. These comparisons assume that each model was trained to reach the same classification accuracy.

## Appendix E Additional Comparisons of Trojans

### Comparison of trojans with location shift (T3 and T4):

The placement of T4 caused for the NN to become instable. We observed that even after more than 2000 epochs, the accuracy could not reach close to 100% as illustrated in Figure A6. This is confirmed by computing negative modified KL divergence values which indicate that the NN model TwT is insufficient to represent the training data. As a consequence, the fluctuation of inefficiency values is larger than in a stable well-trained model. This illustrates that adversaries also face a challenge when choosing the characteristics of embedded trojans in order to conceal them by achieving close to 100% classification accuracy.

### Comparison of trojans embedded in different classes (T3 and T5):

The trojans T3 and T5 are symmetric in terms of their embedding in class P (blue region) or class N (orange region). We observe this symmetry in Figure A7 as the deltas have inverse signs for classes P and N (Δ(*T*6/*P*) > Δ(*T*6/*N*) and Δ(*T*7/*P*) < Δ(*T*7/*N*) except for layer 0). While the chosen locations for embedding trojans T3 and T5 can yield close to 100% classification accuracy, the models heavily depend on the NN initialization. Therefore, we did not compare the inefficiency across the two trojans.

### Comparison of trojans with varying shape (T6 and T7):

Figure A8 summarizes the delta between modified KL divergence values of models TwoT and models TwT for the trojans T6 and T7 of different shapes (circle and square) and embedded into both P and N classes. All deltas are positive for both classes and for all layers except from the last layer (T6, Class N: *δ* = −0.047 and T7, Class P: *δ* = −0.284). Following Figure 6, these values imply that the trojan is in both classes. The values in the last layer indicate that the model TwT had a hard time accurately encoding the trojan.

It is difficult to infer anything about the trojan shapes from Figure A8(right) because the delta curves depend on the very many possible spatial partitions of the 2D space to classify training data points accurately. Nonetheless, one can infer from Figure A8 (right) that the spatial partition allocated for the class P in a model TwT T6 is larger than the in a model TwT T7 (i.e., $\hat{D_{KL}}\left(\text{TwoT}(6)/P\right)-\hat{D_{KL}}(TwT(6)/P)>\hat{D_{KL}}(\text{TwoT}(7)/P)-\hat{D_{KL}}(TwT(7)/P)$. This can be visually confirmed for class P (blue) in Figure A8(left) as the model TwT T6 occupies a larger partition than in the model TwT T7 (i.e., blue area is larger in Figure A8 left middle then in left bottom). A similar inference can be derived for class N as $\hat{D_{KL}}(\text{TwoT}(6)/N)-\hat{D_{KL}}(TwT(6)/N)<\hat{D_{KL}}(\text{TwoT}(7)/N)-\hat{D_{KL}}(TwT(7)/N)$.
